# Genomic analysis of a *Neisseria meningitidis* patient isolate causing a false-positive result in the Abbott Alinity m STI, Sweden: a case report

**DOI:** 10.1128/asmcr.00143-25

**Published:** 2025-09-30

**Authors:** Björn Herrmann, Andreas Sälléber, Rene Kaden

**Affiliations:** 1 Section of Clinical Microbioloby, Department Medical Sciences, Uppsala University 8097, Uppsala, Sweden; 2 Department of Clinical Microbiology, Uppsala University Hospital 59561, Uppsala, Sweden; 3 Department of Clinical Microbiology, Hospital of Västmanland, Västerås, Sweden; 4 Science for Life Laboratory, Uppsala University 8097, Uppsala, Sweden; Vanderbilt University Medical Center, Nashville, Tennessee, USA

**Keywords:** *Neisseria meningitidis*, *Neisseria gonorrhoeae*, Abbott Alinity m STI, diagnostics, PCR

## Abstract

**Background:**

Diagnostics of sexually transmitted infections, such as *Neisseria gonorrhoeae* (NG), are often performed using high-volume test systems, often in combination with other targets. This report examines the occurrence of two false-positive NG results from pharyngeal samples in the Abbott Alinity m STI assay, cleared by the Food and Drug Administration.

**Case Summary:**

Analysis of pharyngeal samples from two men who have sex with men was positive for NG in the Abbott Alinity m STI assay, but negative upon confirmation testing with the Cepheid Xpert CT/NG assay. A pharyngeal culture isolate was obtained from one of the two men. The isolate tested negative for being NG in four different commercial nucleic acid amplification tests. A laboratory-developed PCR based on two targets gave inconclusive results, with positive detection for the *opa* gene but no detection for the *porA* gene. The isolate was identified as *Neisseria meningitidis* (NM) in MALDI-TOF analysis. Whole-genome sequencing revealed 322 diverse virulence genes, of which 314 were typical for NM and 8 for NG. Phylogenetic analysis showed a taxonomic delineation for NM.

**Conclusion:**

Confirmatory tests with alternative targets are essential for accurate analysis of NG, especially in low-prevalence settings. Evaluation of diagnostic tools to ensure accurate detection of NG is also needed.

## INTRODUCTION

Infections with *Neisseria gonorrhoeae* (NG) and *Chlamydia trachomatis* are often diagnosed by commercial high-volume test systems. False species identification may cause unnecessary antibiotic treatment and, for sexually transmitted infections, unwarranted partner notification, social stigma, or relationship termination. In low-prevalence populations such as Sweden, which currently has a gonorrhea incidence of approximately 35 cases per 100,000 inhabitants (1), confirmatory tests are strongly recommended for the detection of NG (2). When using nucleic acid amplification tests to detect NG, the positive predictive value of the test should exceed 90%. Otherwise, a confirmatory test, based on an alternative target sequence with high specificity, should be used. This is crucial for the identification of NG from extragenital samples, as several other *Neisseria* spp. are commensals in the mucosa, particularly the throat. For several years, commercial NG assays have been approved, and the Food and Drug Administration (FDA) has permitted the testing of urine, vaginal, and cervical samples, while extragenital samples have been excluded. Despite this shortcoming, many European laboratories have been using these assays for extragenital samples after in-house validation. Recently, several commercial NG tests have been FDA cleared for throat and rectal samples; among these is the Abbott Alinity m STI assay (denoted as “Alinity” in the text; Abbott Laboratories, Chicago, IL, USA) (3). We, therefore, aimed to investigate two cases of NG-positive results in the Alinity assay, which displayed negative results in a confirmatory test assay (Xpert CT/NG, Cepheid, Sunnyvale, CA, USA) in a local laboratory in Västerås, Sweden.

## CASE PRESENTATION

Routine analysis of pharyngeal samples from two men who have sex with male patients currently receiving pre-exposure prophylaxis (PrEP) for HIV was positive for NG in the Alinity assay, but negative using confirmatory testing with the Xpert CT/NG assay. Both patients had a history of sexually transmitted infections (including successfully treated anogenital gonorrhoeae), but were asymptomatic at the time of sampling. Testing was performed in accordance with local PrEP guidelines. The patients had no known contact with each other but admitted to high-risk sexual behavior. Alinity results for rectal and urine samples from both patients were repeatedly negative. Four repeated pharyngeal samples for each patient, during a 3-month period in early 2023, yielded identical results positive results using the Alinity assay and negative results using the Xpert CT/NG assay. CT values obtained using the Alinity assay were 32.3, 32.2, 26.6, and 26.3 for the first patient (patient A) and 29.1, 37.6, 24.2, and 36.2 for the second patient (patient B). Results were reported as inconclusive, and further investigations were conducted to determine the underlying cause of the discrepancies.

Pharyngeal swab cultures were collected contemporaneously with all but the first PCR samples for each patient. Samples were cultured using GC agar (Becton Dickinson, Franklin Lakes, NJ, USA) with added IsoVitaleX and V-C-N-T inhibitor. One culture from patient A yielded pure growth of suspected colonies of NG, which were further analyzed. No isolate from patient B could be further analyzed as no culture yielded any suspected NG isolates.

MALDI-ToF MS (VITEK MS 3.2 BioMérieux, Marcy-l’Étoile, France, and MALDI Biotyper 3.1, MBT Compass Library Revision L, 9607 MSP, Bruker Daltonics, Billerica, MA, USA) identified the isolate, designated UG2300056, as *Neisseria meningitidis* (NM) with high confidence scores (VITEK MS score 99.9; Bruker biotyper score 2.03). The cultured isolate was retested using both the Alinity assay and the Xpert CT/NG assay to verify the PCR results. Several isolated colonies were suspended in an Alinity m multi-collect sample tube, and for the Xpert CT/NG assay, a sterile sample tube containing 1 mL of nuclease-free water was used, before being treated according to the manufacturer’s instructions. The Alinity assay yielded repeated positive results with low CT values (16.2, 17.3, and 16.3), whereas the Xpert CT/NG assay yielded negative results. The strain from patient A was analyzed using three additional commercial assays, and all results were negative for NG (Table 1). A laboratory-developed PCR, based on two targets, was inconclusive (positive for the *opa* gene and negative for the *porA* gene) (4).

**TABLE 1:** Results of diagnostic tests for the isolate

Method	NG target	Approval status	Result from isolate
Abbott Alinity m STI	*opa*	FDA cleared^*a*^	Positive
Cepheid GeneXpert	Not publicly available	FDA cleared^*a*^	Negative
Roche Cobas 6800 CT/NG	DR9 region, two targets	FDA cleared^*a*^	Negative
Hologic Aptima Combo2	*16S rRNA*	FDA cleared^*a*^	Negative
Hologic Aptima GC	*16S rRNA*	FDA cleared^*b*^	Negative
Becton Dickinson BD COR	Not publicly available	FDA cleared^*b*^	Negative
Laboratory-developed test used as confirmation PCR	*opa PCR*	Not applicable	Positive
	*porA PCR*		Negative

^*a*^ FDA cleared, also for extragenital samples.

^*b*^ FDA cleared, but not for extragenital samples.

Antibiotic susceptibility testing was performed using MIC-gradient tests (ETEST, BioMérieux, France) according to the manufacturer’s instructions. Applying the EUCAST 2025 MIC breakpoints, the strain was susceptible to ceftriaxone (MIC 0.004 mg/L), benzylpenicillin (MIC 0.25 mg/L, beta-lactamase negative), cefotaxime (MIC 0.016 mg/L), tetracycline (MIC 0.25 mg/L), and ciprofloxacin (MIC 0.004 mg/L).

Whole-genome sequencing of the strain was performed with Nanopore technology using the VolTRAX library preparation instrument without multiplexing, and the genome was deposited in GenBank (CP171264.1).

**Fig 1:**
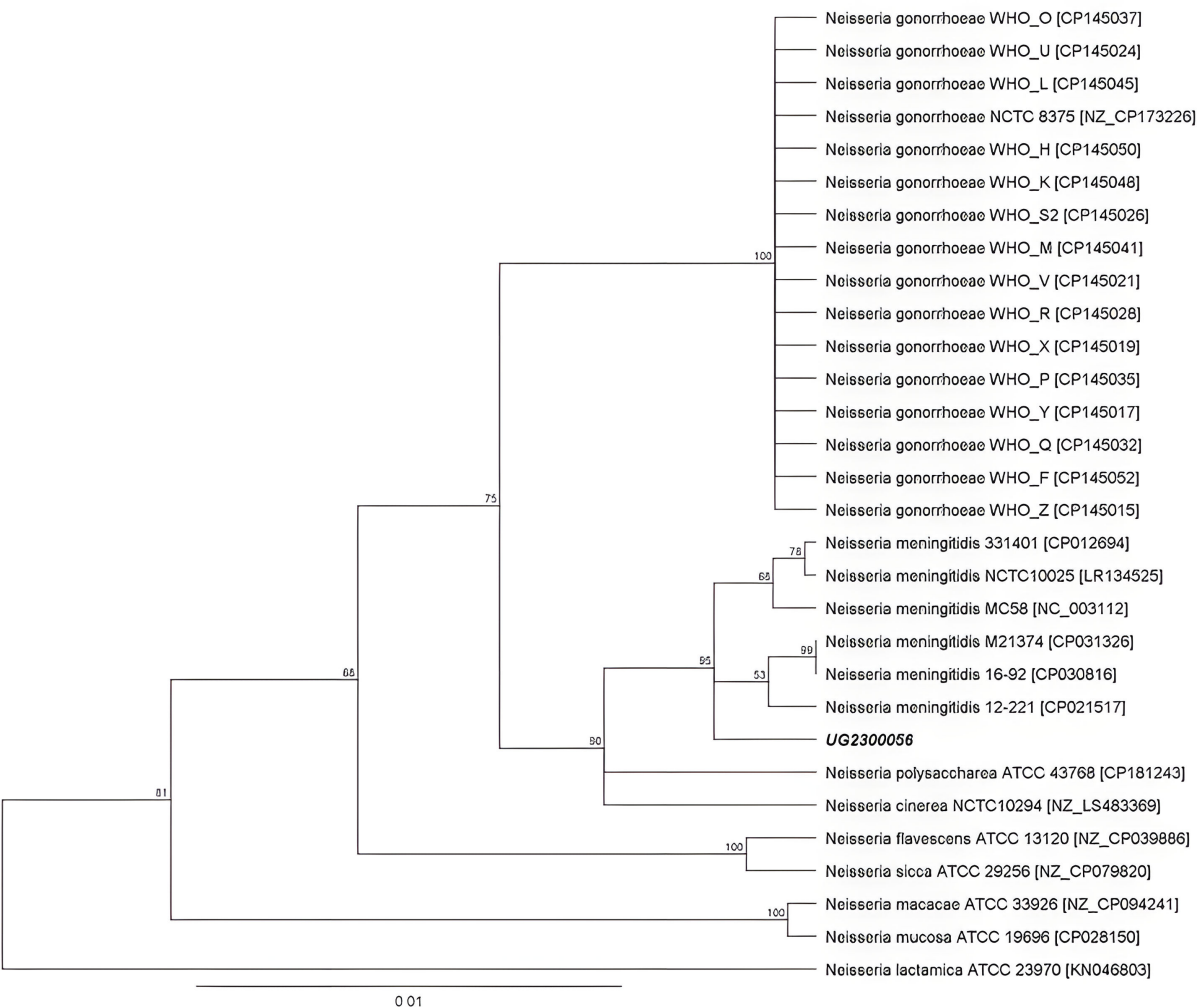
Phylogenetic 16S rRNA tree of isolate UG2300056 and 29 related *Neisseria* spp. strains.

For taxonomic classification and to confirm that a pure culture was sequenced, the *16S rRNA* genes were analyzed (Fig. 1). The four *16S rRNA* genes that were present in the strain showed high similarity to NM NCTC 10025 (range 99.24%–99.45%).

The comparison of the sequences for *opa* and *porA* genes gave inconclusive results (Table 2).

**TABLE 2:** Genomic homology of the examined isolate compared to *N. gonorrhoeae* NCTC 8375^T^ and *N. meningitidis* NCTC10025^T^

	*N. gonorrhoeae*	*N. meningitidis*
16S rDNA	98.5%	99.34%
*opa* gene	98.9%	80.9%
*porA* gene	89.1%	92.8%
ANI	82.3%	90.6%

**Fig 2:**
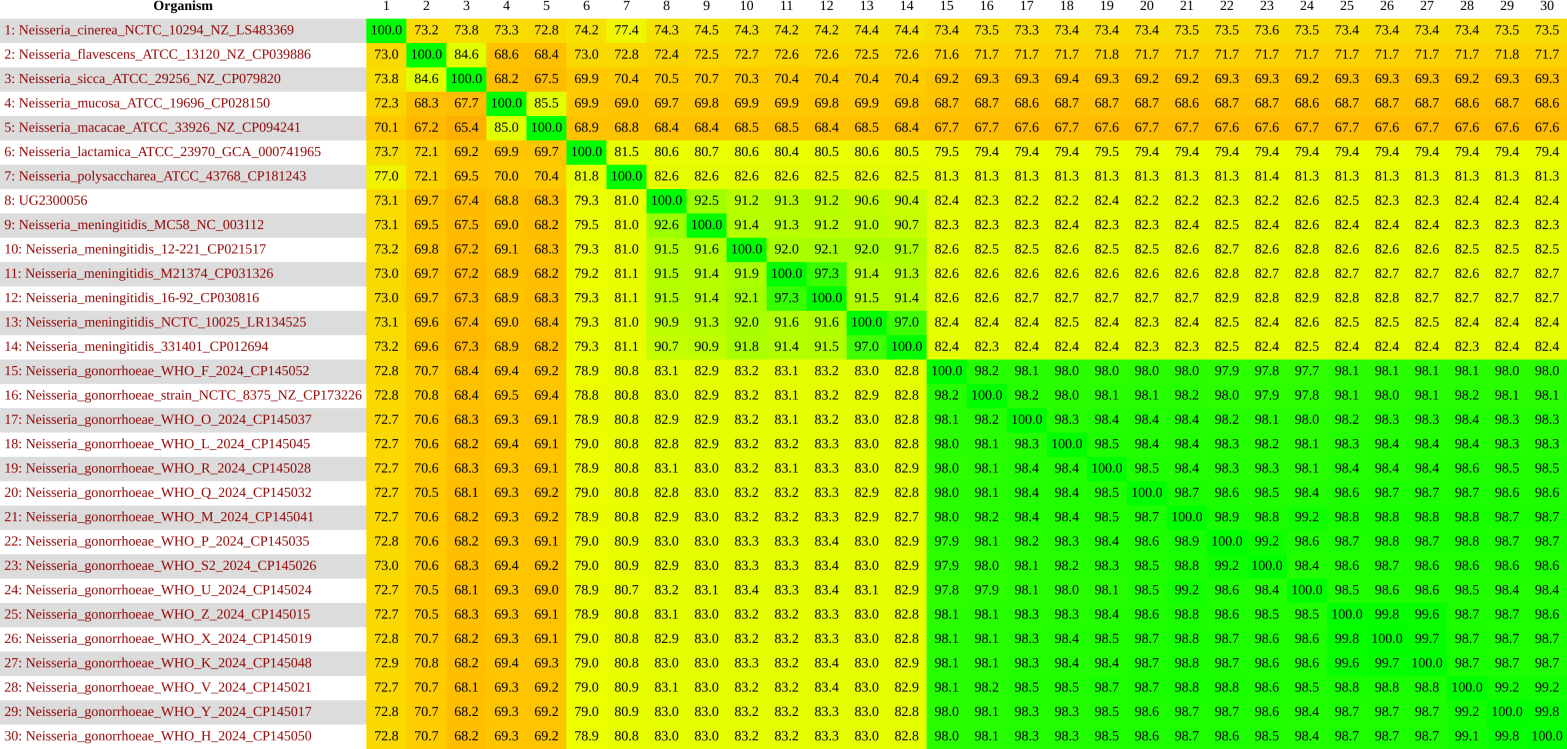
ANI analysis of isolate UG2300056 and 29 related *Neisseria* spp. strains.

Average nucleotide identity (ANI) analysis was conducted to better understand the taxonomic relationship of the strain. The genome of the examined isolate differed by 9.4% from NM and 17.7% from NG type strains (Fig. 2). The results of different genetic analyses showed that the isolate had a taxonomic delineation for NM.

The genome is presented as a single chromosome with a size of 2,437,067 bases and a GC content of 51.1%, while the genome of NM and NG consists of 2,186,098 (51.9% GC) and 2,171,755 (52.6% GC) bases, respectively. The bigger genome of the study isolate was not explained by the presence of plasmids. However, it could result in a higher content of functional genes on the chromosome. Based on the results of Pathofinder (5), the bacterial strain was predicted as human pathogen, of which 314 genes were derived from NM and 8 from NG. Many virulence genes specifically matched the NM serogroup C FAM18.

Ribosomal Multilocus Sequence Typing (MLST) was used for microbial typing (6) and resulted in a species determination of NM with a support of 95%. MLST assigned the isolate to ST11506 within NM (7).

Although infection with NG could not be confirmed, the attending physician for patient A opted to treat with a single 1-g dose of ceftriaxone. Follow-up samples collected 30 days post-treatment tested negative by PCR. The patient remained asymptomatic throughout the investigation period. Patient B did not attend any subsequent visits and was ultimately lost to follow-up.

## DISCUSSION

It has previously been shown for *Neisseria* spp. that molecular assays have had shortcomings in differentiating between NG and NM (8), especially NM associated with urethritis (9).

In our case study, the target in the Alinity assay is the *opa* gene. The specificity of the Alinity assay for NG was tested on 16 strains from 11 *Neisseria* spp. (including 5 NM strains of different serotypes) before FDA clearance (10). The *opa* gene has commonly been used for NG detection, often in a duplex PCR with *porA* gene for confirmation of first-line detection with commercial assays. When using the *opa* gene together with the *porA* gene, it has been shown that the *opa* gene more commonly yields a positive result than the *porA* gene, especially for pharyngeal samples (11). It may also be seen as insufficient to consider *opa*-negative *porA*-positive strains as true NG. Irrespective of how these targets are used for confirmation, it may be problematic (11).

We performed *16S rRNA* gene analysis since it is commonly used for bacterial taxonomy. However, it is not a recommended marker for distinguishing *Neisseria* species (12) as the 16S DNA homology within all species of this genus is high, especially between the two most clinically relevant species, NG and NM, which share 98.6% of their *16S rRNA*.

Our whole-genome analysis indicates that the isolate is a NM strain that acquired additional genes from NG. The species of the genus *Neisseria* are known to share genes by transformation, homologous recombination, and multiple types of mobile genetic elements. Genome analysis identified *penA* mutations I312M and V316T, both commonly found in mosaic *penA* strains of type 60.001 and associated with resistance to beta-lactams and quinolones (13). However, epistasis, in which phenotypic resistance is dependent on complex interactions of multiple mutant genes (14), may explain why our isolate displayed sensitivity toward all antibiotics tested. Furthermore, a beta-lactam resistance gene with a similarity of 94.9% to *penA* (NCBI accession AF515059) was found. Despite the genomic distance, the phenotype was predicted to be resistant (9).

### Conclusion

The detailed analysis indicates that the current strain is genetically closest to NM but has also incorporated genetic elements from NG, leading to false-positive results in the Alinity m STI assay targeting *opa*, and in the *opa* gene in the laboratory developed duplex PCR.

This case emphasizes the necessity for confirmatory testing with two different target sequences. Evaluation of diagnostic tools to ensure accurate detection of NG is also needed. Therefore, it is imperative to thoroughly analyze unexpected results.

## Data Availability

The whole-genome sequence of the NM isolate is available in NCBI GenBank under the accession number CP171264.1.
